# Subamolide B Isolated from Medicinal Plant *Cinnamomum subavenium* Induces Cytotoxicity in Human Cutaneous Squamous Cell Carcinoma Cells through Mitochondrial and CHOP-Dependent Cell Death Pathways

**DOI:** 10.1155/2013/630415

**Published:** 2013-03-13

**Authors:** Shu-Yi Yang, Hui-Min Wang, Tai-Wen Wu, Yi-Ju Chen, Jeng-Jer Shieh, Ju-Hwa Lin, Tsing-Fen Ho, Ren-Jie Luo, Chung-Yi Chen, Chia-Che Chang

**Affiliations:** ^1^Institute of Biomedical Sciences, National Chung Hsing University, 250 Kuo-Kuang Road, Taichung 40227, Taiwan; ^2^Department of Fragrance and Cosmetic Science, Kaohsiung Medical University, Kaohsiung 80708, Taiwan; ^3^Department of Dermatology, Taichung Veterans General Hospital, Taichung 40705, Taiwan; ^4^Department of Medicine, National Yang-Ming University School of Medicine, Taipei 11221, Taiwan; ^5^Department of Biological Science and Technology, China Medical University, Taichung 40402, Taiwan; ^6^Department of Medical Laboratory Science and Biotechnology, Central Taiwan University of Science and Technology, Taichung 40601, Taiwan; ^7^School of Medical and Health Sciences, Fooyin University, Kaohsiung 83102, Taiwan; ^8^Agricultural Biotechnology Center, National Chung Hsing University, Taichung 40227, Taiwan; ^9^Graduate Institute of Basic Medical Science, China Medical University, Taichung 40402, Taiwan

## Abstract

Subamolide B is a butanolide isolated from *Cinnamomum subavenium*, a medicinal plant traditionally used to treat various ailments including carcinomatous swelling. We herein reported for the first time that subamolide B potently induced cytotoxicity against diverse human skin cancer cell lines while sparing nonmalignant cells. Mechanistic studies on human cutaneous squamous cell carcinoma (SCC) cell line SCC12 highlighted the involvement of apoptosis in subamolide B-induced cytotoxicity, as evidenced by the activation of caspases-8, -9, -4, and -3, the increase in annexin V-positive population, and the partial restoration of cell viability by cotreatment with the pan-caspase inhibitor z-VAD-fmk. Additionally, subamolide B evoked cell death pathways mediated by FasL/Fas, mitochondria, and endoplasmic reticulum (ER) stress, as supported by subamolide B-induced FasL upregulation, BCL-2 suppression/cytosolic release of cytochrome c, and UPR activation/CHOP upregulation, respectively. Noteworthy, ectopic expression of c-FLIP_L_ or dominant-negative mutant of FADD failed to impair subamolide B-induced cytotoxicity, whereas BCL-2 overexpression or CHOP depletion greatly rescued subamolide B-stimulated cells. Collectively, these results underscored the central role of mitochondrial and CHOP-mediated cell death pathways in subamolide B-induced cytotoxicity. Our findings further implicate the potential of subamolide B for cutaneous SCC therapy or as a lead compound for developing novel chemotherapeutic agents.

## 1. Introduction 

In North America, around half of all cancers occur on the skin [1]. In USA, nonmelanoma skin cancers, including cutaneous squamous cell carcinoma (SCC) and basal cell carcinoma (BCC), have shown a doubling rate of incidence from 1994 to 1996 and were reported to affect nearly 3.69 million patients in 2008 [2]. SCC represents the second most common skin cancer [3]. In contrast to other nonmelanoma skin cancers, SCC carries a significant risk of metastasis and accounts for the majority of the several thousand deaths attributable to nonmelanoma skin cancer each year [4]. Exposure to ultraviolet radiation (UV) is believed to be a major etiological cofactor in the development of cutaneous SCC [5]. Surgical treatment remains as the standard of care for nonmelanoma skin cancers and is successful for the majority of patients. Additionally, other traditional and new regimens of nonsurgical approaches, including photodynamic therapy and topical immunomodulators, provide a good cosmetic and therapeutic success especially in those of poor surgery candidate. Imiquimod is the most commonly used topical drug for the treatment of actinic keratosis and superficial BCC and SCC [6]. However, patients with aggressive or advanced diseases frequently suffer from high rates of treatment failure, morbidity, and mortality by traditional methods of chemotherapy or radiotherapy. Thus, development of novel therapeutics for effective treatment of these difficult or aggressive cancers is in urgent demand.

Subamolide B [(3E,4R,5R)-3-tetradecylidene-4-hydroxy-5-methoxy-5-methyl-butanolide] (Supplementary Figure 1 available online at http://dx.doi.org/10.1155/2013/630415) is a butanolide isolated from *Cinnamomum subavenium* Miq. (Lauraceae), a medium-sized evergreen tree distributed in Burma, Cambodia, central and southern parts of China, Indonesia, Malaysia, and Taiwan [7]. Notably, *C. subavenium* has been used as a traditional Chinese medicine for treating a broad range of illnesses, including carcinomatous swelling, chest pain, abdominal pain, stomachache, diarrhea, hernia, nausea, vomiting, and rheumatism [8]. Not until recently are the bioactive butanolides of *C. subavenium* isolated and functionally characterized. Subamolide A, an isomer of subamolide B, has been reported to induce apoptosis in human colon adenocarcinoma cell line SW480 and human urothelial carcinoma cell line NTUB1 in addition to acting as an inhibitor of human tyrosinase [7–9]. Furthermore, an in vitro antimelanoma activity has been assigned to subamolide E, another butanolide isolated from *C. subavenium* [10, 11]. As to the bioactivity of subamolide B, the only report so far is its ability to induce apoptosis in SW480 cells [7].

The tumoricidal activity of most, if not all, chemotherapeutics is attributed to their effect to induce cancer cell apoptosis [12, 13]. Apoptotic signals are transmitted through extrinsic (death receptor) or intrinsic (mitochondria) pathways to induce caspase activation, the cardinal hallmark of apoptosis. Engagement of the death ligand FasL to its cognate receptors Fas leads to cytoplasmic binding to the death domain (DD) of the adaptor molecule Fas-associated death domain (FADD), which in turn recruits procaspase-8 through interaction with their death effector domains (DEDs) to form the death-inducing signaling complex (DISC) for caspase-8 activation [13]. Conversely, c-FLIP competes with caspase-8 for binding to the DISC complex but is devoid of caspase activity, thus precluding caspase-8 activation in a dominant negative manner [14]. Mitochondrial apoptosis pathway is controlled at the level of the mitochondrial outer membrane integrity, which is tightly regulated by members of BCL-2 protein family such as prosurvival BCL-2 and BCL-xL in addition to proapoptotic BAX and BAK [15]. In particular, a decrease in the ratio of prosurvival to proapoptotic BCL-2 family proteins leads to the disruption of the mitochondrial outer membrane and consequent cytosolic release of cytochrome c to form the apoptosome complex with Apaf-1 for caspase-9 activation [16, 17]. Activation of caspases-8 and -9 in turn initiates a series of caspase cascade for caspase-3 activation, which accounts for the biochemical and cellular features of apoptosis [17].

In addition to mitochondria, endoplasmic reticulum (ER) is another organelle involved in cell death initiation [18]. Unfolded or misfolded proteins accumulated in the ER lumen lead to ER stress, which in turn activates the unfolded protein response (UPR) signaling pathways consisting of three canonical branches, namely, IRE1, PERK, and ATF6, to induce transcriptional upregulation of chaperon proteins like GRP78 to reestablish homeostasis in the ER [19]. Under irremediable ER stress, the adaptive nature of the UPR signaling switches to the initiation of apoptotic program, predominantly mediated by transcriptional induction of the proapoptotic transcription factor CHOP/GADD153 (C/EBP homologous protein/Growth arrest and DNA damage-inducible gene 153) [20, 21]. CHOP promotes cell death in part through downregulating prosurvival BCL-2 and/or upregulating proapoptotic BIM [22, 23], consequently leading to the initiation of mitochondrial apoptosis pathway.

Although subamolide B's proapoptotic activity on SW480 cells was shown, how subamolide B induces apoptosis was not interrogated in that report [7]. Likewise, the cytotoxic effect of subamolide B on skin cancer cells has never been addressed previously. For those reasons, in this study we aimed to elucidate the cytotoxic effect of subamolide B on human skin cancer cell lines and also its underlying mechanism. We herein provide evidence that subamolide B potently induces cell death of both melanoma and nonmelanoma skin cancer cell lines while sparing nonmalignant cells, and subamolide B-induced cytotoxicity mainly involves the activation of mitochondrial cell death pathway as well as the induction of cytotoxic endoplasmic reticulum response. Our results therefore provide a novel mechanistic insight into the cytotoxic action of subamolide B but also implicate the potential of using subamolide B as an antiskin cancer biologic drug or a lead compound for developing novel anticancer therapeutics.

## 2. Materials and Methods

### 2.1. Purification of Subamolide B from *C. subavenium*

The air-dried stems of *C. subavenium* (8.0 kg) were extracted with methanol (80 L x 6) at room temperature and a methanol extract (202.5 g) was obtained upon concentration under reduced pressure. The methanol extract, suspended in H_2_O (1 L), was partitioned with CHCl_3_(2 L x 5) to give fractions soluble in CHCl_3_ (123.5 g) and H_2_O (74.1 g). The CHCl_3_-soluble fraction (123.5 g) was chromatographed over silica gel (800 g, 70~230 mesh) using n-hexane/ethyl acetate/methanol mixtures as eluents to produce five fractions. Part of fraction 2 (9.31 g) was subjected to silica gel chromatography, eluted with n-hexane-ethyl acetate (10 : 1), and then enriched gradually with ethyl acetate to furnish five fractions (2-1~2-5). Subsequently, fraction 2-4 (1.31 g) was subjected to silica gel chromatography, eluted with n-hexane-ethyl acetate (40 : 1), and enriched gradually with ethyl acetate to obtain four fractions (2-4-1~2-4-4). Fraction 2-4-2 (1.06 g) was further separated using silica gel column chromatography (eluted with n-hexane-ethyl acetate (10 : 1)) and preparative TLC (n-hexane-ethyl acetate (30 : 1)) to give subamolide B (42 mg). The structure of subamolide B was then identified by spectroscopic analysis (^1^H, ^13^C NMR, COSY, NOESY, HMBC, HMQC) with purity higher than 95%.

### 2.2. Drugs

Purified subamolide B was stocked as 10 mM in DMSO. Thapsigargin (Calbiochem, Darmstadt, Germany) was prepared as 1 mM stock solution in DMSO. z-VAD-fmk (N-benzyloxycarbonyl-Val-Ala-Asp-fluoro-methyl-ketone) (Bachem, Torrance, CA, USA) was stocked in DMSO at a concentration of 20 mM. All chemicals in stock solutions were stored in aliquots at −20°C until use.

### 2.3. Cell Culture

The human cutaneous squamous cell carcinoma cell line SCC12 (kindly provided by Prof. Xiaoqi Wang, Northwestern University, USA) [24] was grown in DMEM/F-12 (1 : 1) medium. The human malignant melanoma cell line A375 (ATCC number: CRL-1619), human epidermoid carcinoma cell line A-431 (ATCC number: CRL-1555), and HEK293T cells (ATCC number: CRL-11268) were grown in DMEM medium, whereas the human basal cell carcinoma cell line BCC-1/KMC [25] was cultured in RPMI1640 medium. Normal human skin fibroblast cell lines CCD-966SK (ATCC number: CRL-1881) and WS1 (ATCC number: CRL-1502) as well as normal human lung fibroblasts IMR-90 (ATCC number: CCL-186), MRC-5 (ATCC number: CCL-171), and WI-38 (ATCC number: CCL-75) were all obtained from BCRC (Bioresource Collection and Research Center, Hsinchu, Taiwan) and grown in MEM medium. All media were purchased from Gibco BRL (Grand Island, NY, USA) and were supplemented with 10% heat-inactivated fetal bovine serum (Gibco BRL, Grand Island, NY, USA), 100 U/ml penicillin, and 100 mg/mL streptomycin. Cells were allowed to grow at 37°C in a humidified, 5% CO_2_ atmosphere.

### 2.4. Cell Viability Determination

The effect of subamolide B on cell viability was mostly evaluated using CellTiter 96 AQ_ueous_ Non-Radioactive Cell Proliferation Assay (MTS assay) kit (Promega, Madison, WI, USA) as previously described [26]. Alternatively, propidium iodide (PI) exclusion assay was employed to determine cell viability [27]. In brief, 4 × 10^5^ SCC12 cells were seeded onto 6-well dishes and were subjected to 48 h treatment with subamolide B. Cells were then trypsinized, collected by centrifugation, and washed twice with PBS. Subsequently, cells were stained with 10 *μ*g/ml of PI in PBS for 15 min in the dark, followed by flow cytometry analysis to determine the levels of PI incorporation.

### 2.5. Colony Formation Assay

The effect of subamolide B on the long-term survival of SCC12 cells was assessed using colony formation assay in accordance with the reported procedure [26]. Briefly, 5 × 10^2^ SCC12 cells treated for 24 h with subamolide B (20 *μ*M) were seeded per well of 6-well culture dishes and were allowed to grow in drug-free media for 10~14 d to form colonies. The numbers of colonies were then revealed by staining with 1% crystal violet solution in 30% ethanol. The colonies composed of 50 or more cells were counted under microscopy. At least three independent assays were performed to calculate the numbers of colonies for statistical analysis.

### 2.6. Annexin-V/Propidium Iodide (PI) Dual Staining Assay

Annexin V/PI dual staining assay was employed to determine the involvement of apoptosis in subamolide B-induced cell death, using ApoTarget Annexin-V-FITC Apoptosis kit (Biosource, Camarillo, CA, USA) as described previously [28]. If required, the pan-caspase inhibitor z-VAD-fmk (20 *μ*M) was added to SCC12 cells 2 h before subamolide B treatment.

### 2.7. Immunoblot Analysis

Immunoblotting was performed as previously described [26]. Antibodies against BCL-2, CHOP, eIF2*α*, Ser51-phosphorylated eIF2*α*, GRP78, HA epitope, IRE1, and PARP were all obtained from Cell Signaling Technology (Danvers, MA, USA). The antibody detecting Ser724- phosphorylated IRE1 was purchased from Epitomics (Burlingame, CA, USA). Anti-*β*-tubulin, serving as the control for equal loading, was obtained from Sigma-Aldrich (St. Louis, MO, USA).

### 2.8. Reverse Transcription-Polymerase Chain Reaction (RT-PCR)

Total RNA extraction, 1st strand cDNA preparation, and SYBR green-based real-time PCR were performed as reported previously [26]. The sequence of primer pairs used for semiquantitative PCR are listed as follows: FasL forward, 5′-GGTCCATGCCTCTGGAATGG-3′ and FasL reverse, 5′-CACATCTGCCCAGTAGTGCA-3′; Fas forward, 5′-CAGAACTTGGAAGGCCTGCATC-3′ and Fas reverse, 5′-TCTGTTCTGCTGTGTCTTGGAC-3′; CHOP forward 5′-CAACTGCAGAGATGGCAGCTG-3′ and CHOP reverse, 5′-ACTGATGCTCCCAATTGTTCATG-3′; XBP-1 forward 5′-CCTTGTAGTTGAGAACCAGG-3′ and XBP-1 reverse, 5′-GGGGCTTGGTATATATGTGG-3′; *β*-actin forward, 5′-TCACCCACACTGTGCCCATCTACGA-3′ and *β*-actin reverse, 5′-CAGCGGAACCGCTCATTGCCAATGG-3′. For quantitative real-time RT-PCR, the following primer pairs were used: TRAIL forward, 5′-GACCTGCGTGCTGATCGTG-3′ and TRAIL reverse, 5′-TGTCCTGCATCTGCTTCAGCT-3′; DR4 forward, 5′-TCCACAAAGAATCAGGCAATGGAC-3′ and DR4 reverse, 5′-TGCAACAACAGACAATCAGCACAG-3′; DR5 forward, 5′-AGTCAGAGCATCTGCTGGAAC-3′ and DR5 reverse, 5′-AGCACTGTCTCAGAGTCTCAG-3′; TBP (TATA-binding protein) forward, 5′-CACGAACCACGGCACTGATT-3′ and TBP reverse, 5′-TTTTCTTGCTGCCAGTCTGGAC-3′. The levels of mRNA expression of *genes-in-interest* were normalized to that of *TBP*. Final results were presented as fold induction of the ratio of the genes-in-interest mRNA copy numbers to *TBP* mRNA copy numbers after subamolide B stimulation compared to that of drug-untreated control.

### 2.9. Luciferase Reporter Assay

The ATF6 activity reporter plasmid p5xATF6-GL3 (Addgene plasmid 11976) carries five ATF6 response elements in tandem to drive the expression of firefly luciferase. The human *CHOP * promoter, which encompasses the region between −947 and +30 (+1 is the transcriptional start site) was PCR-amplified from the genomic DNA of peripheral blood mononuclear cells using the primer pair 5′-GGTACCGGTGAAACGTAGTCTCGCTCTG-3′ (forward) and 5′-AAGCTTCAGTGCCTTAGACTTAAGTCTCTG-3′ (reverse) [29, 30]. The 978-bp fragment was cloned into the luciferase reporter plasmid pGL4.18 vector (Promega, Madison, WI, USA) to generate the human *CHOP * promoter reporter plasmid (pCHOP-Luc). Luciferase activity assay was performed using Dual-Luciferase Reporter assay kit (Promega, Madison, WI, USA) as reported previously [31]. Luciferase activity was normalized to *Renilla* luciferase activity, and final results were expressed as fold induction of the luciferase activity after subamolide B stimulation compared to that of drug-untreated control.

### 2.10. RNA Interference

CHOP knockdown was achieved by RNA interference-mediated suppression mechanism by targeting the mRNA sequence 5′-CAGAACCAGCAGAGGTCACAA-3′ of the human *CHOP* gene (genbank accession no. NM_004083). The complementary oligonucleotides containing the target sequence were annealed and subcloned into the pMKO retroviral vector (kindly provided by Dr. Alan Y.-L. Lee, National Health Research Institute, Taiwan) to generate the CHOP siRNA-expressing plasmid, shCHOP.

### 2.11. Construction of pBabe.puro-Based Expression Plasmids

The BCL-2-expressing plasmid pBabe-BCL2 was generated by subcloning the EcoRI-digested cDNA fragment containing the open reading frame (ORF) of human BCL-2 from the plasmid 3336pcDNA3 Bcl-2 (Addgene plasmid 8768) into the EcoRI site of pBabe-puro retroviral vector [30]. To construct the vector expressing the dominant-negative mutant of human FADD (dnFADD), the region containing the DD domain but devoid of the DED domain was amplified by PCR using the primer pair 5′-ACCGGTGCCGCGCCTGGGGAAGAAGAC-3′ (forward) and 5′-ATCAGGACGCTTCGGAGGTAG-3′ (reverse). The PCR-amplified fragment was then subcloned into a modified pBabe-puro vector allowing to place an upstream in-frame HA epitope (pBabe-HA), yielding the plasmid pBabe-HA-dnFADD. Similarly, the ORF of the long isoform of c-FLIP (c-FLIP_L_) was PCR-amplified by the primers 5′-ACCGGTTCTGCTGAAGTCATCCATCAGGTTG-3′ (forward) and 5′-CTCGAGTTATGTGTAGGAGAGGATAAG- TTTCTTTC-3′ (reverse), followed by insertion of the PCR-amplified fragment into the pBabe-HA vector to generate the plasmid pBabe-HA-c-FLIP_L_.

### 2.12. pBabe.puro- and pMKO-Derived Retroviral Particle Production and Infection

Production and infection of retroviral vectors expressing HA-dnFADD, HA-cFLIP_L_, BCL-2, and shCHOP in HEK-293T and SCC12 cells, respectively, were conducted according to the reported protocol established in our laboratory [31].

### 2.13. Statistical Analysis

All data were expressed as a means ± standard error of mean (SEM) from at least three independent experiments. Differences between groups were examined for statistical significance using Student's *t*-test. A *P* value lower than 0.05 was used as the minimum criteria for statistical significance.

## 3. Results

### 3.1. Subamolide B Selectively Induces Cytotoxicity in Diverse Human Skin Cancer Cell Lines While Sparing Nonmalignant Cells

**Figure 1:**
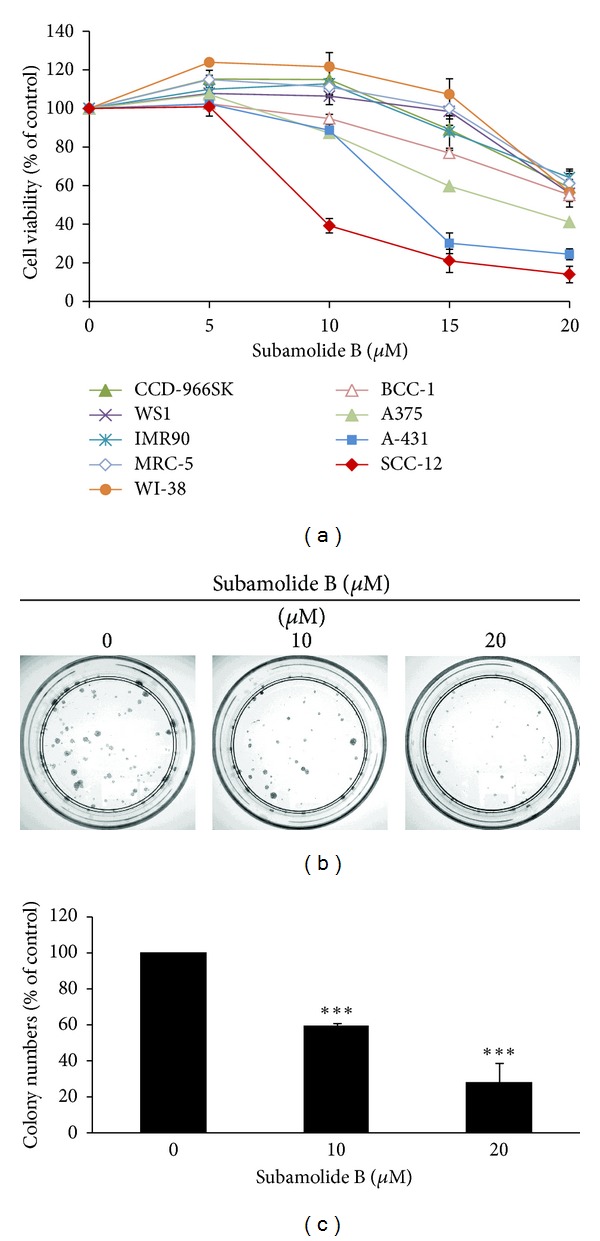
Cytotoxic effect of subamolide B on human skin cancer cell lines. (a) Dose-dependent cytotoxicity of subamolide B. Human skin cancer cell lines, including A375 (melanoma), A-431 (epidermoid carcinoma), BCC-1/KMC (basal cell carcinoma) and SCC12 (cutaneous squamous cell carcinoma), normal human skin fibroblast cell lines CCD-966SK and WS1, and normal human lung fibroblast cell lines IMR-90, MRC-5, and WI-38 were treated with graded doses of subamolide B (0~20 *μ*M), followed by cell viability determination *via* MTS assay as described in Section 2. (b) Dose-dependent repression of SCC12 colony formation by subamolide B. SCC 12 cells were treated with subamolide B (0~20 *μ*M) for 24 h and then allowed to grow in drug-free media to form colonies. 14 days later, the extent of subamolide B-mediated repression of SCC12 colony formation was evaluated by the numbers of colonies grown on culture dishes. Shown here are the representative images from at least three independent experiments. (c) Quantitative analysis of subamolide B-mediated repression of SCC12 colony formation. The numbers of colonies shown in (b) were counted and the results were presented as the percentage of drug-treated groups compared to the drug-free controls. ***P < 0.001.

 We first evaluated the cytotoxic effect of subamolide B on various human skin cancer cell lines, including A375 (melanoma), A-431 (epidermoid carcinoma), BCC-1/KMC (basal cell carcinoma), and SCC12 (cutaneous squamous cell carcinoma). As shown in Figure 1(a), subamolide B markedly reduced the viability of A375, A-431, and SCC12 cells in a dose-dependent manner, with SCC12 cells more sensitive to subamolide B-induced cytotoxicity (IC_50_: 9.12 *μ*M) than A375 (IC_50_: 17.59 *μ*M) and A-431 cells (IC_50_: 13.30 *μ*M) (Table 1). Instead, BCC-1/KMC cells were rather insensitive to subamolide B-elicited cytotoxicity (IC_50_:  >20 *μ*M) (Figure 1(a); Table 1). It is noteworthy that subamolide B's cytotoxic effect was relatively limited on all nonmalignant cell lines tested, including human skin fibroblast cell lines CCD-966SK and WS1 as well as human lung fibroblasts IMR-90, MRC-5, and WI-38 (Figure 1(a); Table 1). The cytotoxic activity of subamolide B was further substantiated by its ability to suppress the colony-forming capacity of SCC12 cells (Figure 1(b)). Particularly, treatment with 10 *μ*M and 20 *μ*M of subamolide B repressed the levels of SCC12 colony formation to 58.93 ± 2.40% and 28.20 ± 10.87% of the drug-untreated control, respectively (*P* < 0.001) (Figure 1(c)). Taken together, these results illustrated the cytotoxic effect of subamolide B on three of the four human skin cancer cell lines examined with limited toxicity to nonmalignant cells. Given that SCC12 cells were particularly susceptible to subamolide B-induced cytotoxicity, this cell line was used as the cellular model for further mechanistic inquiry on subamolide B's cytotoxic action.

### 3.2. Subamolide B-Induced Cytotoxicity Involves Caspase Activation

**Figure 2:**
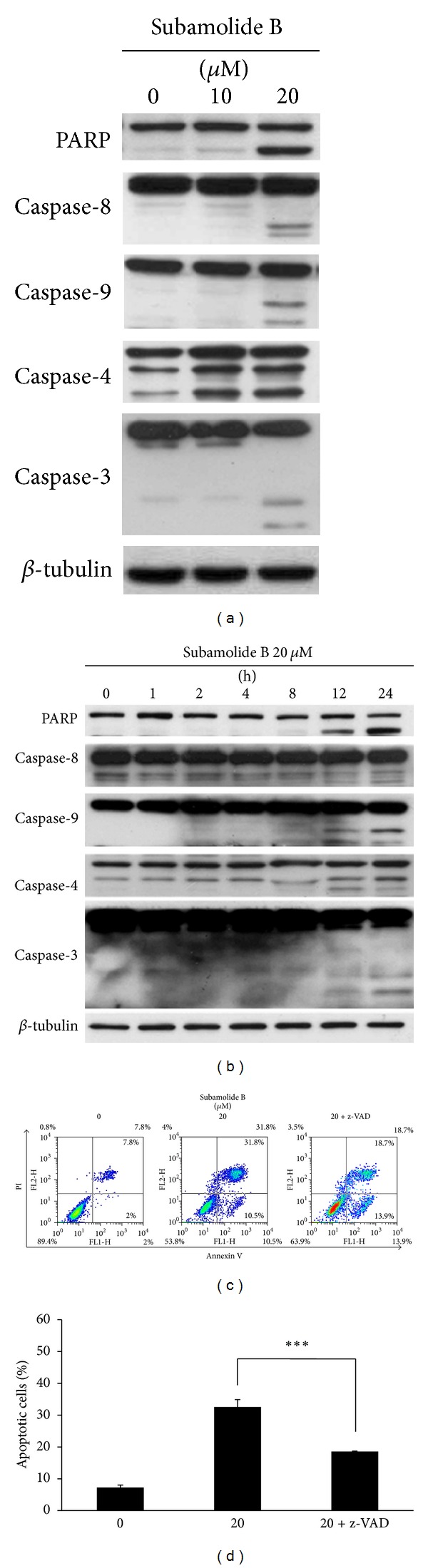
Apoptosis is partly responsible for subamolide B-induced SCC12 cell death. ((a), (b)) Subamolide B induces dose- and time-dependent proteolytic processing/activation of caspases-8, -9, -4, and -3. SCC12 cells were treated with subamolide B (0~20 *μ*M) for 24 h (a) or treated with 20 *μ*M of subamolide B for indicated length of time (b), followed by immunoblot analysis of caspase activation revealed by the status of proteolytic processing of PARP and procaspases-8, -9, -4, and -3. *β*-tubulin was used as the loading control. ((c), (d)) Caspase activities and hence apoptosis induction represent a mechanism of action of subamolide B-induced SCC12 cell death. Subamolide B (20 *μ*M) was used to treat SCC12 cells for 24 h without or with cotreatment of the pan-caspase inhibitor z-VAD-fmk (20 *μ*M). Cells were then revealed by Annexin-V/Propidium iodide (PI) double staining using flow cytometry analysis. The levels of apoptotic cells were indicated as the percentage of all cell populations as shown in (d). ***P < 0.001.

 We next examined whether subamolide B evokes SCC12 cell death through the induction of apoptosis. To this end, we first asked whether subamolide B elicits caspase activation, a cardinal hallmark of apoptosis. It is noteworthy that subamolide B induced the cleavage of PARP, suggesting the activation of caspases (Figure 2(a)). Detailed analyses indicated that subamolide B treatment led to a marked proteolytic processing of procaspases-8, -9, -4, and -3, indicating that these caspases are activated. Furthermore, kinetic study revealed a marked increase of cleaved PARP and processed caspases-8, -9, -4, -3 at 12 h following subamolide B treatment (Figure 2(b)). To further validate the caspase dependence of subamolide B-induced cytotoxicity, SCC12 cells were pretreated for 2 h with the pan-caspase inhibitor z-VAD-fmk (20 *μ*M) and then were subjected to treatment with 20 *μ*M of subamolide B. It is clear to note that z-VAD-fmk treatment suppressed the level of apoptotic cells upon subamolide B stimulation (Figure 2(c)). Specifically, the level of subamolide B-induced apoptotic cells was reduced from 32.55 ± 3.65% to 18.6 ± 0.42% when cotreated with z-VAD-fmk (*P* < 0.001) (Figure 2(d)). Taken together, these data established that induction of apoptosis represents one of the modes of subamolide B's cytotoxic action.

### 3.3. Subamolide B Activates Both Extrinsic and Intrinsic Cell Death Pathways

**Figure 3:**
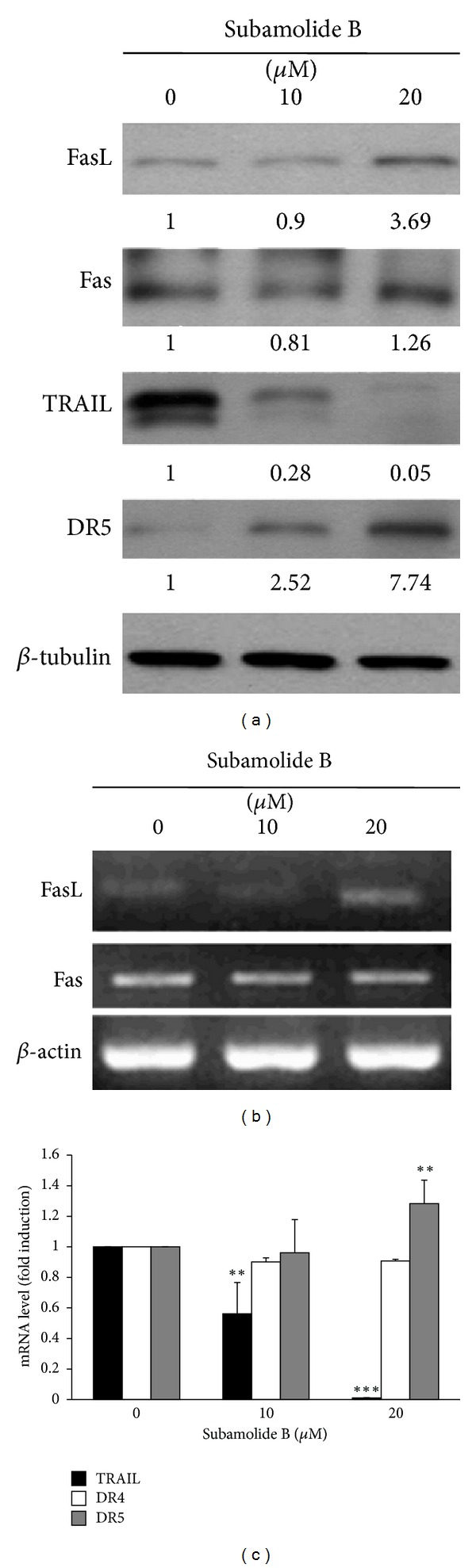
Subamolide B appears to engage the FasL/Fas cell death pathway. (a) Subamolide B upregulates FasL. SCC12 cells were treated with 0~20 *μ*M of subamolide B for 24 h, followed by immunoblotting to monitor the levels of various death receptor ligands and their cognate receptors, including FasL/Fas and TRAIL/DR5. *β*-tubulin was used as the loading control. (b) Subamolide B treatment increases the mRNA levels of FasL but not Fas. The mRNA expression levels of FasL and Fas in SCC12 cells treated with subamolide B (0~20 *μ*M) for 24 h were monitored by semiquantitative RT-PCR analysis. *β*-actin was used as the loading control. (c) Effects of subamolide B on the mRNA expression levels of TRAIL, DR4, and DR5. SCC12 cells treated with subamolide B as described in (b) were subjected to quantitative real-time RT-PCR analysis for the mRNA expression levels of TRAIL and the two cognate TRAIL receptors DR4 and DR5. It is noted that subamolide B decreases the mRNA levels of TRAIL while increasing those of DR5 and barely modulates DR4 expression. The mRNA levels are presented as the ratio of *genes-in-interest* mRNA levels normalized to TATA box-binding protein (*TBP*) mRNA levels after subamolide B treatment to that of drug-untreated controls. **P < 0.01; ***P < 0.001.

 The proteolytic processing/activation of caspase-8 and caspase-9 highly implicated the initiation of the extrinsic and intrinsic apoptotic pathways, respectively. We therefore tried to elucidate the upstream molecular events leading to the activation of caspases-8 and -9 upon subamolide B stimulation. The effect of subamolide B on the expression of various death ligands and their cognate receptors was first evaluated. Our results indicated that both the protein and mRNA levels of FasL were clearly increased after treatment with 20 *μ*M of subamolide B, whereas the expression of its receptor Fas was barely affected (Figures 3(a), and 3(b)). Kinetic analysis further revealed a time-dependent increase of FasL protein expression induced by subamolide B (Supplementary Figure 2). Furthermore, subamolide B downregulated TRAIL while upregulating its receptor DR5 in a dose-dependent manner (Figures 3(a) and 3(c)). Conversely, quantitative real-time RT-PCR analysis revealed subamolide B's limited effect on the mRNA expression of another TRAIL receptor, DR4 (Figure 3(c)). It is therefore likely that the FasL/Fas-mediated extrinsic apoptotic pathway was elicited by subamolide B to induce caspase-8 activation.

**Figure 4:**
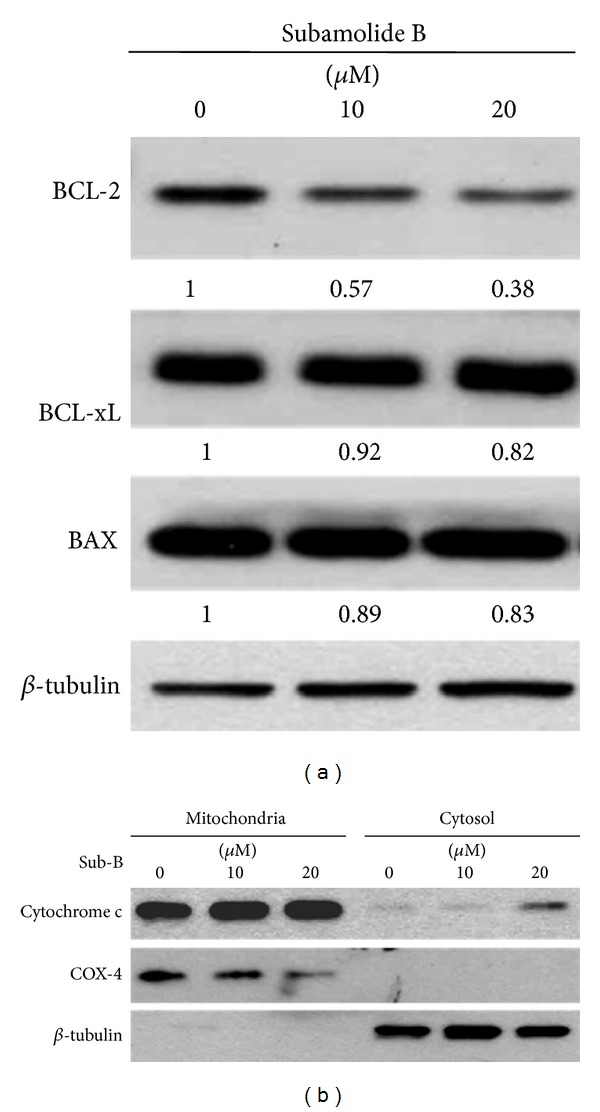
Subamolide B activates the intrinsic (mitochondrial) apoptosis pathway. (a) Subamolide B dose-dependently downregulates BCL-2. SCC12 cells treated with subamolide B (0~20 *μ*M) for 24 h were assessed for the levels of prosurvival BCL-2, BCL-xL, and proapoptotic BAX using immunoblot analysis. *β*-tubulin was used as the loading control. The density of each subamolide B-treated immunoblot signal was presented as the ratio to that of drug-untreated controls. (b) Subamolide B induces cytochrome c release to the cytosol. SCC12 cells treated with subamolide B (0~20 *μ*M) for 24 h were fractionated into the mitochondrial and cytosolic fractions. The levels of cytochrome c in each fraction were then revealed by immunoblotting. The levels of COX-4 and *β*-tubulin were the loading controls for the mitochondrial and cytosolic fractions, respectively. Sub-B: subamolide B.

 We next addressed how subamolide B engages the intrinsic (mitochondrial) apoptotic pathway to induce caspase-9 activation. Given that members of BCL-2 protein family are primarily responsible for initiating mitochondria-mediated apoptosis, the effect of subamolide B on the expression of BCL-2 family proteins was examined. It is noteworthy that subamolide B evoked a dose- and time-dependent reduction in the level of prosurvival BCL-2, whereas it barely modulates the levels of prosurvival BCL-xL or the prodeath BAX (Figure 4(a); Supplementary Figure 2). This resultant alteration of BCL-2 family protein expression apparently lowered the ratio of prosurvival to prodeath BCL-2 family proteins, theoretically causing the disruption of mitochondrial membrane integrity and consequent cytosolic release of cytochrome c to initiate caspase-9 activation. In line with this, a dose-dependent increase of cytochrome c levels in the cytosol was elicited after subamolide B treatment (Figure 4(b)). Taken together, subamolide B appears to engage the FasL-Fas axis and lower the BCL-2/BAX ratio to activate the extrinsic and intrinsic apoptosis pathways for the consequent activation of caspase-8 and caspase-9, respectively.

### 3.4. Subamolide B Induces ER Stress Response

**Figure 5:**
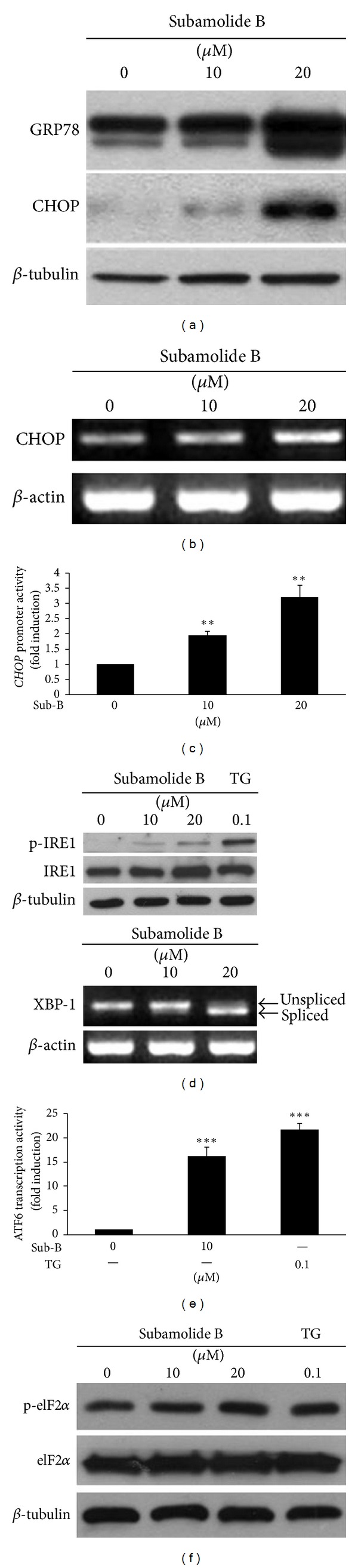
Subamolide B is an ER stress inducer. (a) Subamolide B upregulates signature ER stress markers GRP78 and CHOP. SCC12 cells treated with subamolide B (0~20 *μ*M) for 24 h were subjected to immunoblot analysis for the levels of GRP78 and CHOP. *β*-tubulin was used as the loading control. (b) Subamolide B increases *CHOP* mRNA expression levels. The level of *CHOP* mRNA in SCC12 cells after 24 h treatment with subamolide B (0~20 *μ*M) was assessed by semiquantitative RT-PCR analysis. *β*-actin was used as the loading control. (c) Subamolide B activates the human *CHOP* promoter. SCC12 cells transiently transfected with the human *CHOP* promoter reporter plasmid pCHOP-Luc (−947~+30) were subjected to subamolide treatment (0~20 *μ*M) for 24 h, followed by luciferase activity assay to determine the *CHOP* promoter activity. (d) Subamolide B activates the IRE1 arm of the UPR signaling pathway. SCC12 cells were treated with subamolide B (0~20 *μ*M) or the ER stress inducer thapsigargin (0.1 *μ*M) for 24 h, followed by immunoblotting and semiquantitative RT-PCR analyses for the levels of Ser724-phosphorylated IRE1/total IRE1 (upper panel) and the splicing of *XBP1* mRNA (lower panel), respectively. The levels of *β*-tubulin and *β*-actin were the respective loading controls for immunoblotting and semiquantitative RT-PCR analyses. (e) Subamolide B induces ATF6 activation. SCC12 cells were transiently transfected with the ATF6 transcriptional activity reporter plasmid (p5xATF6-GL3) and then under 24 h treatment with subamolide B (0, 10 *μ*M) or thapsigargin (0.1 *μ*M). ATF6 transcriptional activity was then evaluated by luciferase activity assay. (f) Subamolide B engages the PERK branch of the UPR signaling pathway. SCC12 cells after 24 h treatment with subamolide B (0~20 *μ*M) or thapsigargin (0.1 *μ*M) were subjected to immunoblotting for the levels of PERK-mediated Ser51 phosphorylation of eIF2*α* (p-eIF2*α*) and total eIF2*α*. *β*-tubulin was used as the loading control. The activity of the *CHOP *promoter or ATF6 is presented as the ratio of the luciferase activities after drug treatment to that of drug-untreated controls. **P < 0.01; ***P < 0.001. Sub-B: subamolide B; TG: thapsigargin.

 Activation of caspase-4 is an indicator of a proapoptotic ER stress response [32]. Our finding that subamolide B evoked caspase-4 activation (Figures 2(a) and 2(b)) thus implicated the possible involvement of ER stress in subamolide B-induced cytotoxicity. Indeed, we observed that subamolide B dose-dependently upregulated signature ER stress markers GRP78 and CHOP (Figure 5(a)). Kinetic studies revealed a time-dependent induction of GRP78 by subamolide B while the induction of CHOP peaked at 12 h after subamolide B treatment (Supplementary Figure 2). Additional evidence indicated that subamolide B up-regulated CHOP at the level of transcription, as supported by a dose-dependent induction of both *CHOP* mRNA expression (Figure 5(b)) and the *CHOP* promoter activity (*P* < 0.01) (Figure 5(c)) after treatment with subamolide B. To further validate subamolide B's capacity to induce ER stress, we asked whether ER stress-induced UPR signaling pathways are evoked upon subamolide B stimulation. It is clear to note that subamolide B treatment caused IRE1 autophosphorylation (upper panel) and the splicing of *XBP1* mRNA (lower panel), indicating the activation of the IRE1 branch of UPR signaling (Figure 5(d)). It is also apparent that subamolide B activated the ATF6 arm of the UPR, as revealed by a 16.09 ± 2.06 fold induction of ATF6 transcriptional activity when treated with 10 *μ*M of subamolide B (*P* < 0.001) (Figure 5(e)). Lastly, subamolide B was found to induce PERK-mediated phosphorylation of eIF2*α* to the level comparable to that induced by the ER stress inducer thapsigargin, suggesting that subamolide B engages the PERK branch of UPR signaling (Figure 5(f)). Altogether, these results identified subamolide B as an inducer of ER stress and UPR signaling.

### 3.5. Subamolide B-Induced Cytotoxicity Requires BCL-2 and CHOP

**Figure 6:**
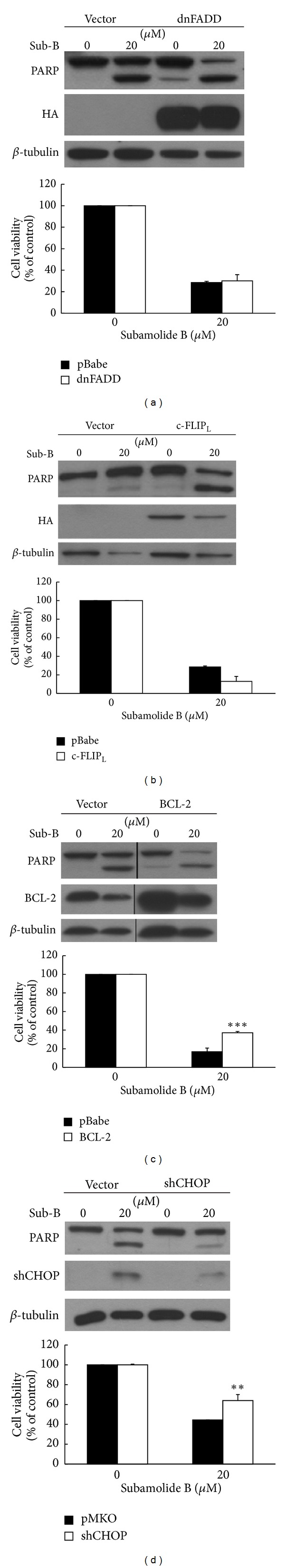
Subamolide B induces SCC12 cell death mainly through activating mitochondrial cell death pathway and cytotoxic ER stress response. ((a), (b)) The FasL/Fas cell death pathway is not required for subamolide B-induced cytotoxicity. SCC12 cells were stably infected with the pBabe vector alone or the vectors expressing a dominant-negative form of FADD (dnFADD) (a) or c-FLIPL (b) to block the function of the FasL/Fas cell death pathway. These stable clones were then treated with 20 *μ*M of subamolide B for 24 h to monitor the status of PARP cleavage in addition to enforced expression of dnFADD or c-FLIP_L_ (left panel), or for 48 h to determine cell viability using PI exclusion assay (right panel). (c) Mitochondrial cell death pathway is involved in subamolide B-induced cytotoxicity. SCC12 cells stably carrying the pBabe vector alone or the vector overexpressing BCL-2 to impair the activation of mitochondrial cell death pathway were treated with subamolide B (20 *μ*M) for 24 h and 48 h to evaluate the effect of enforced BCL-2 expression on subamolide B-induced PARP cleavage (left panel) and cytotoxicity (right panel), respectively. (d) Involvement of CHOP-mediated ER stress cell death pathway in subamolide B-induced cytotoxicity. SCC12 cells were stably infected with the pMKO vector alone or the vector expressing CHOP-specific siRNA (shCHOP) to inhibit ER stress-induced cell death. These stable clones were then treated with subamolide B (20 *μ*M) for 24 h and 48 h to evaluate the effect of CHOP knockdown on subamolide B-induced PARP cleavage (left panel) and cytotoxicity (right panel), respectively. **P < 0.01; ***P < 0.001. Sub-B: subamolide B.

 Given that subamolide B activates the extrinsic, intrinsic, and ER stress cell death pathways, we further elucidated the functional significance of these pathways in subamolide-induced cytotoxicity. To clarify the role of the extrinsic cell death pathway, SCC12 cells were stably infected with the pBabe retroviral vectors expressing HA-tagged c-FLIP_L_ or a dominant-negative form of FADD (dnFADD), which preserves the DD domain for binding to the Fas receptor but is devoid of the DED domain required for interacting with caspase-8, in order to functionally impair the extrinsic pathway. The established dnFADD stable clones (Figure 6(a)) and c-FLIP_L_ stable clones (Figure 6(b)) were then treated with 20 *μ*M of subamolide B for 48 h and their viability was determined thereafter. To our surprise, subamolide B effectively induced PARP cleavage and cell death despite ectopic expression of dnFADD or c-FLIP_L_, thus illustrating a dispensable role of the extrinsic cell death pathway in subamolide B-induced cytotoxicity.

 Next, SCC12 clones stably overexpressing BCL-2 were established to evaluate the role of the intrinsic (mitochondrial) cell death pathway in subamolide B-induced cytotoxicity. Consistent with previous observations, subamolide B treatment suppressed BCL-2 levels and also led to a marked induction of PARP cleavage and cell death in vector-infected control cells (Figure 6(c)). In contrast, enforced BCL-2 expression markedly reduced the extents of PARP cleavage and cell death elicited by subamolide B (Figure 6(c)). These data therefore supported the involvement of the intrinsic cell death pathway in subamolide B-induced cytotoxicity.

 CHOP is regarded as a principal proapoptotic transcriptional factor mediating cytotoxic ER stress responses [20, 21] and, notably, was up-regulated by subamolide B (Figures 5(a)–5(c)). We therefore addressed the role of ER stress cell death pathway by evaluating subamolide B's cytotoxic effect on SCC12 cells whose endogenous CHOP was depleted by its specific shRNA (shCHOP). It is noteworthy that CHOP depletion significantly repressed PARP cleavage and also enhanced the survival of subamolide B-treated cells (Figure 6(d)). Collectively, the evidence that both BCL-2 and CHOP are required for subamolide B-induced cytotoxicity supports the notion that both the mitochondrial and ER stress-mediated cell death represent two fundamental modes of action underlying the cytotoxic effect of subamolide B.

## 4. Discussion

**Figure 7:**
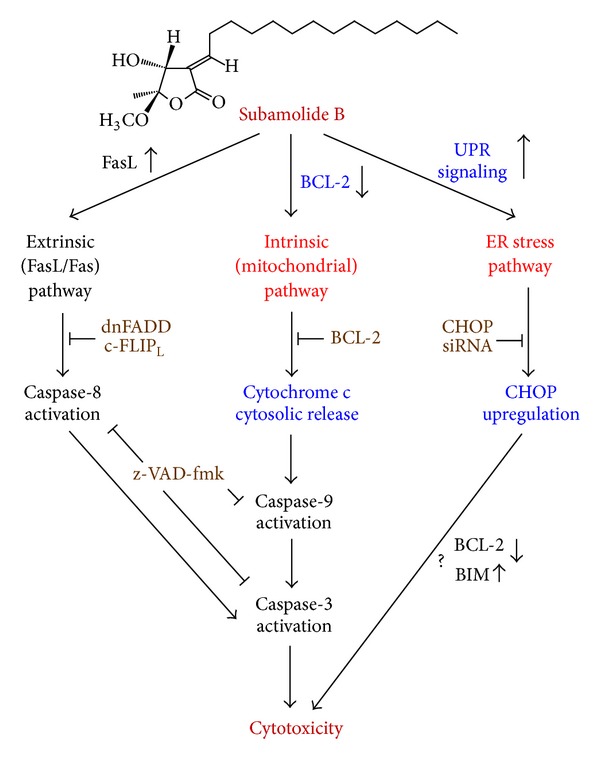
A proposed model for the cytotoxic mechanisms of action of subamolide B. Subamolide B activates the cell death pathways mediated by FasL/Fas, mitochondria, and ER stress, as evidenced by FasL upregulation, BCL-2 downregulation/cytosolic release of cytochrome c, and induction of the UPR signaling pathway/CHOP upregulation, respectively. These cell death pathways lead to the activation of caspase-8, caspase-9, caspase-4, and caspase-3. Caspase activity and thus apoptosis are partially responsible for the cytotoxic activity of subamolide B, as cotreatment with the pan-caspase inhibitor z-VAD-fmk impairs but not abolishes subamolide B-induced cell death. FasL/Fas-mediated pathway appears dispensable for subamolide B's cytotoxic activity, since blockade of this pathway by c-FLIP_L_ or the dominant-negative mutant of FADD (dnFADD) barely affects subamolide B-induced cell death. Conversely, BCL-2 overexpression or CHOP knockdown markedly enhances the survival of subamolide B-treated cells, confirming the requirement of mitochondrial and ER stress cell death pathways for subamolide B-induced cytotoxicity. Whether CHOP contributes to subamolide B's cytotoxic action through suppression of BCL-2 and/or upregulation of BIM requires further validation.

 Natural products isolated from plants or medicinal herbs have been invaluable resources of antineoplastic agents or lead compounds for new drug development [33–35]. Subamolide B is a butanolide isolated from a medicinal plant *Cinnamomum subavenium* traditionally used for treating multiple ailments, including carcinomatous swelling [8]. In this study, the cytotoxic effect and the underlying mechanism of subamolide B were examined. We herein present evidence that subamolide B as a single agent shows effective *in vitro* cytotoxicity against skin cancer cell lines A375 (melanoma), A-431 (epidermoid carcinoma), and SCC12 (cutaneous SCC) while sparing nonmalignant cells (Figure 1). Using SCC12 cell line as our cellular model, we further demonstrated that subamolide B induces activation of caspases-8, -9, -4, and -3 (Figure 2) and stimulates the cell death pathways mediated by FasL/Fas (Figure 3), mitochondria (Figure 4), and ER stress (Figure 5). Notably, functional blockade analysis further elucidates that it is the activation of mitochondrial pathway and cytotoxic ER stress response but not FasL/Fas pathway that is required for subamolide B to induce SCC12 cell death (Figure 6). Based upon these findings, the mechanism of subamolide B's cytotoxic action is delineated (Figure 7). To our best knowledge, this is the first report as to subamolide B-induced cytotoxicity and the underlying mechanism on human cutaneous SCC cells.

 Induction of apoptosis is well recognized as the primary cytotoxic mechanism for the antitumor effect of most chemotherapeutic agents [12, 13]. In line with this, we identified the involvement of apoptosis in subamolide B-induced cell death. This notion is supported by our observation that subamolide B treatment led to the activation of both initiator caspases-8/-9 and effector caspase-3, in addition to the ER stress-associated caspase-4 (Figures 2(a) and 2(b)). Importantly, caspase dependence in subamolide B-induced cytotoxicity was further substantiated by a marked rescue of SCC12 cell viability from subamolide B when the pan-caspase inhibitor z-VAD-fmk was cotreated (Figures 2(c) and 2(d)). On the other hand, it is interesting to note that z-VAD-fmk impaired but failed to abolish subamolide B-induced cell death (Figures 2(c) and 2(d)). These findings therefore illustrate that apoptosis induction is only partly responsible for the cytotoxic mechanism of subamolide B and further implicate that other modes of cell death are involved in subamolide B-elicited cytotoxicity, whose identities need to be elucidated in the future.

 It is noteworthy that most chemotherapeutic agents eradicate cancer cells through initiation of mitochondrial apoptosis pathway [36]. The present study revealed that subamolide B induces the activation of cell death pathways mediated by FasL/Fas, mitochondria, and ER stress, as evidenced by FasL upregulation (Figure 3), BCL-2 downregulation/cytosolic release of cytochrome c (Figure 4), and activation of UPR signaling/CHOP transcriptional upregulation (Figure 5), respectively. However, FasL/Fas pathway was proved dispensable for subamolide-induced cytotoxicity, because enforced expression of dnFADD or c-FLIP_L_ failed to rescue SCC12 cells from subamolide B treatment (Figures 6(a) and 6(b)). Conversely, BCL-2 overexpression (Figure 6(c)) or CHOP depletion (Figure 6(d)) greatly enhanced the survival of subamolide B-treated cells, thus underscoring the involvement of mitochondria- and ER stress-mediated cell death pathways in the cytotoxic mechanism of subamolide B. Importantly, CHOP-elicited cell death is known to involve suppression of prosurvival BCL-2 [22] and/or induction of prodeath BIM [23] culminating in the initiation of mitochondrial cell death pathway [20, 21, 37]. In this context, the modes of cytotoxic action of subamolide B are consistent with those of most current chemotherapeutic agents, which induce cancer cell death predominantly by triggering the mitochondrial pathway while amplifying cell death signal outputs through the extrinsic pathway [36]. Moreover, it is interesting to mention that ER stress-mediated cell death only partly relies on caspase activities [38], thus ostensibly accounting for the partial caspase dependence of subamolide B-induced cytotoxicity (Figures 2(c) and 2(d)).

Data presented here implicates subamolide B as a potential agent for cutaneous SCC therapy. It is noted that imiquimod is currently the most commonly used topical drug for the treatment of superficial BCC and SCC [6]. Intriguingly, we found that SCC12 cells were relatively refractory to imiquimod-induced cytotoxicity, as evidenced by our observation that imiquimod as high as 50 *μ*g/ml reduced SCC12 cell viability only to 65.00 ± 4.32% of the drug-untreated control after 24 h treatment (Supplementary Figure 3). In contrast, subamolide B treatment for 24 h showed an IC_50_ of 6.897 *μ*g/ml (20.27 *μ*M) (Supplementary Figure 3) and thus is much more effective than imiquimod to induce *in vitro* cytotoxicity against SCC12 cells. Still, it should be noted that it remains inconclusive as to whether subamolide B is generally a more potent anticutaneous SCC agent than imiquimod, because we were unable to evaluate the cytotoxicity of subamolide B and imiquimod on additional cutaneous SCC cell lines due to their limited availability. Future studies in preclinical models are therefore necessary to substantiate the potential of subamolide B for cutaneous SCC therapy.

## 5. Conclusion

We provide clear evidence in this study to demonstrate subamolide B-elicited potent cytotoxic effect on various skin cancer cell lines with limited cytotoxicity to nonmalignant cells. Mechanistic investigation in SCC12 cells further defines the activation of mitochondrial cell death pathway and the induction of cytotoxic ER stress response as the fundamental modes of action underlying subamolide B-induced cytotoxicity. These findings unveil mechanistic insights into the cytotoxic action of subamolide B but also implicate the potential of subamolide B as an anticutaneous SCC agent or as a lead compound to develop novel therapeutic agents for cancers including cutaneous SCC.

## Supplementary Material

Supplemental Figure 1: Chemical structure of subamolide B.
Supplemental Figure 2: Kinectic analysis of component molecules involved in the extrinsic, intrinsic and ER stress cell death pathways upon subamolide B stimulation.
Supplemental Figure 3: SCC12 cells were resistant to imiquimod-induced cytotoxicity.

## Figures and Tables

**Table 1:** IC_50_* (*μ*M) of subamolide B for human cell lines examined in this study.

Skin cancer cell lines				Normal skin fibroblasts		Normal lung fibroblasts		
A375	A-431	BCC-1	SCC12	CCD-966SK	WS1	IMR-90	MRC-5	WI-38
17.59	13.30	>20	9.12	>20	>20	>20	>20	>20

*IC_50_ is defined as the dosage of subamolide B capable to reduce cell viability by 50% after 48 h treatment.
