# Supplementary material for: Subamolide B Isolated from Medicinal Plant Cinnamomum subavenium Induces Cytotoxicity in Human Cutaneous Squamous Cell Carcinoma Cells through Mitochondrial and CHOP-Dependent Cell Death Pathways
Source: Evid Based Complement Alternat Med. 2013 Mar 13;2013:630415. doi: 10.1155/2013/630415 (PMC3610371; doi:10.1155/2013/630415)

Supplementary Figure Legends (Yang et al.)

Supplementary Figure 1. Chemical structure of subamolide B. The structure of subamolide B [(3*E*,4*R*,5*R*)-3-tetradecylidene-4-hydroxy-5-methoxy-5-methylbutanolide] is presented.

Supplementary Figure 2. Kinectic analysis of component molecules involved in the extrinsic, intrinsic and ER stress cell death pathways upon subamolide B stimulation. SCC12 cells were treated with subamolide B (20 μM) for 24 h, and the levels of proteins involved in the extrinsic (FasL, Fas), intrinsic (BCL-2, BAX) and ER stress (GRP78, CHOP) pathways were determined at indicated time points by immunoblotting. β-tubulin was used as the loading control.

Supplementary Figure 3. SCC12 cells were resistant to imiquimod-induced cytotoxicity. SCC12 cells were treated with increasing doses of subamolide B (0~20 μM) (**A**) or imiquimod (0~50 μg/ml) (**B**) for 24 h, and the viability of imiquimod-treated cells was evaluated thereafter. It is noted that 20 μM (6.897 μg/ml) of subamolide B reduced the viability of SCC12 cells to 50.47±5.89% compared to the drug-untreated control, whereas 65.00±4.32% of SCC12 cells were still viable after treatment with 50 μg/ml of imiquimod.

Supplementary Figure 1 (Yang et al.)

Subamolide B ($C_{20}H_{36}O_4$; MW: 340.2614)

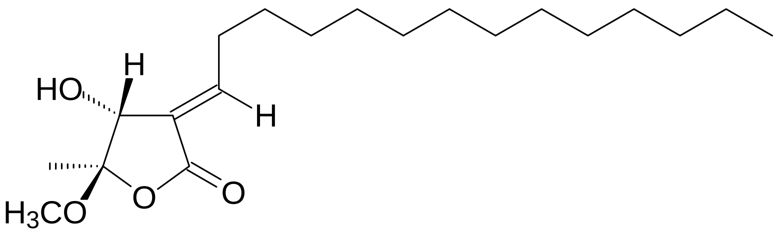

Supplementary Figure 2 (Yang et al.)

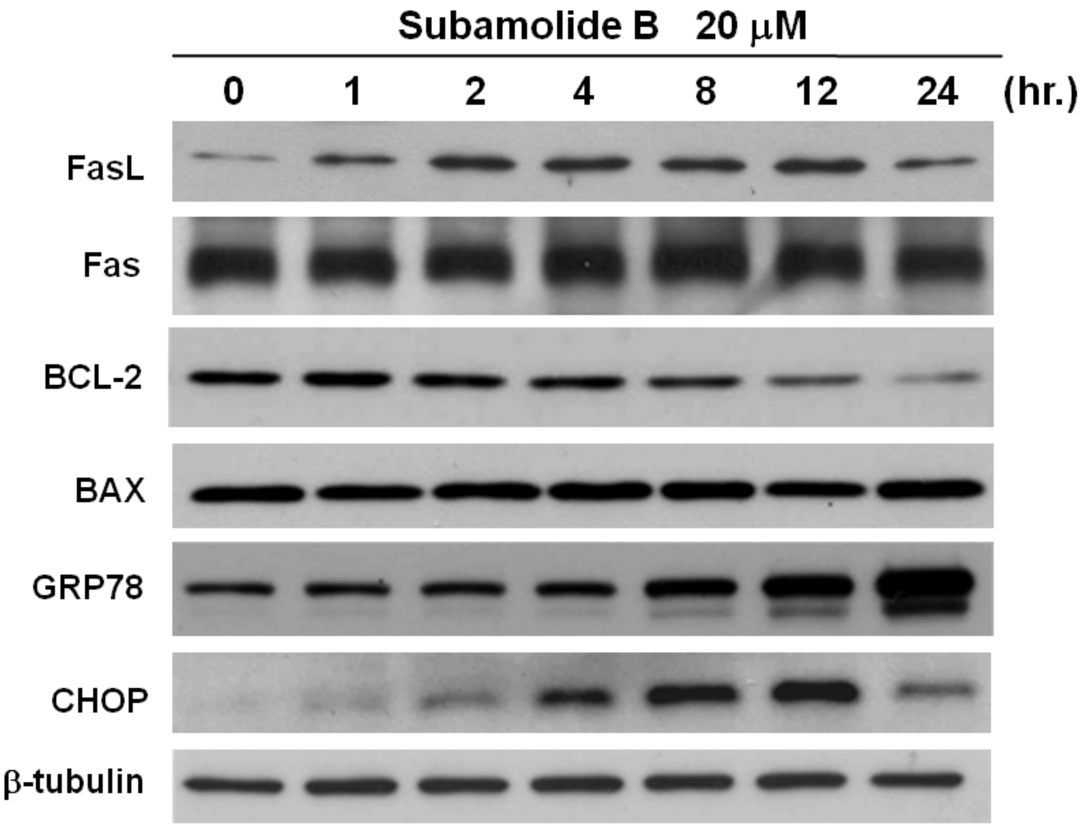

Supplementary Figure 3 (Yang et al.)

A

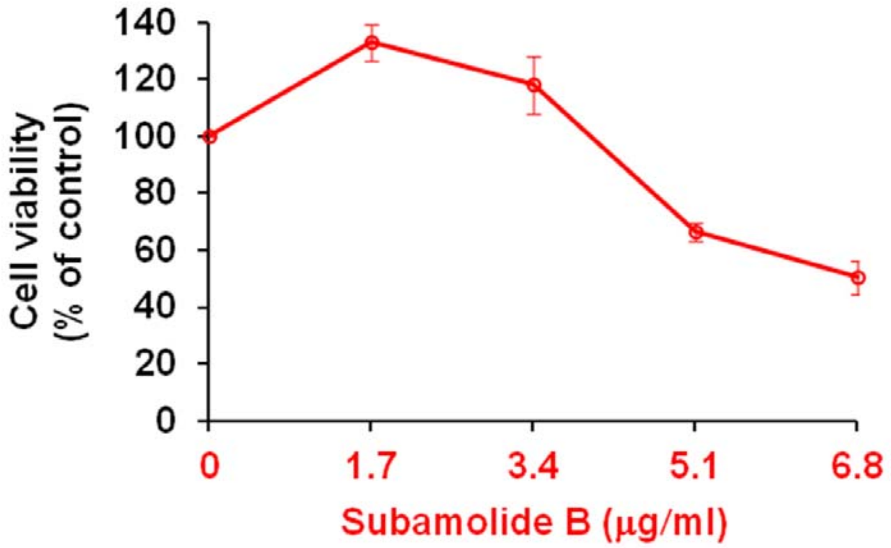

B

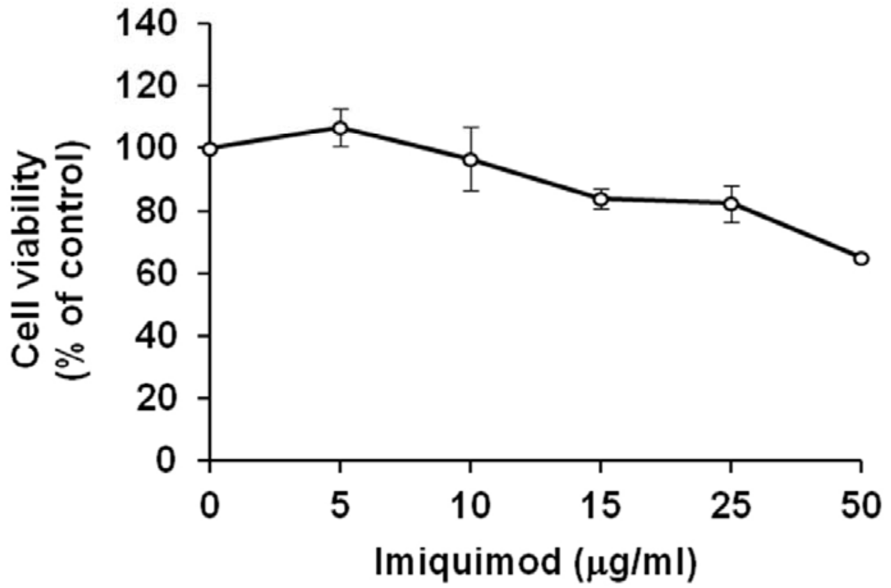

Supplement: Supplementary file 1 — Supplemental Figure 1: Chemical structure of subamolide B. Supplemental Figure 2: Kinectic analysis of component molecules involved in the extrinsic, intrinsic and ER stress cell death pathways upon subamolide B stimulation. Supplemental Figure 3: SCC12 cells were resistant to imiquimod-induced cytotoxicity. [file 630415.f1.pdf]
